# GLP‐1 Agonist Attenuates Nicotine Reward‐Related Behavior by Regulating the Prepro‐Orexin in the Hypothalamus and Prepro‐Glucagon in the Nucleus Tractus Solitarius

**DOI:** 10.1155/bn/1193259

**Published:** 2026-06-17

**Authors:** M. Rahmadi, C. Ardianto, R. Rodsiri, D. Anggraini, A. P. S. A. Firdaussy, I. N. B. Sumartha, A. D. Nurhan

**Affiliations:** ^1^ Department of Pharmacy Practice, Faculty of Pharmacy, Universitas Airlangga, Surabaya, Indonesia, unair.ac.id; ^2^ Biomedical Pharmacy Research Group, Faculty of Pharmacy, Universitas Airlangga, Surabaya, Indonesia, unair.ac.id; ^3^ Department of Pharmacology and Physiology, Faculty of Pharmaceutical Sciences, Chulalongkorn University, Bangkok, Thailand, chula.ac.th; ^4^ Jeffrey Cheah Sunway Medical School, Faculty of Medical and Life Sciences, Sunway University, Sunway City, Malaysia, sunway.edu.my; ^5^ School of Pharmacy, College of Pharmacy, Taipei Medical University, Taipei, Taiwan, tmu.edu.tw

**Keywords:** GLP-1 agonist, liraglutide, nicotine addiction, tobacco addiction, tobacco control

## Abstract

Tobacco consumption has significantly increased globally, contributing to various degenerative diseases such as lung cancer and cardiovascular disorders. Nicotine, the primary addictive component in tobacco, exerts its effects by stimulating nicotinic acetylcholine receptors (nAChRs), leading to excessive dopamine release in the ventral tegmental area (VTA) and nucleus accumbens (NAc), which underlies its rewarding and addictive properties. Additionally, nicotine enhances orexin activity in the hypothalamus and prepro‐glucagon expression in the nucleus tractus solitarius (NTS), further reinforcing addictive behaviors. This study is aimed at investigating the effects of liraglutide, a GLP‐1 receptor agonist, on nicotine‐induced reward. Male mice strain ddy were divided into four groups: control, nicotine, nicotine + liraglutide (50 *μ*g/kg), and nicotine + liraglutide (100 *μ*g/kg). The conditioned place preference (CPP) test was used to evaluate nicotine′s rewarding effects, whereas RT‐PCR assessed the expression of prepro‐orexin and prepro‐glucagon mRNA in the hypothalamus and NTS, respectively. The results confirmed that nicotine administration (0.5 mg/kg) significantly increased CPP scores, indicating enhanced reward‐related behaviors. Correspondingly, nicotine elevated prepro‐orexin and prepro‐glucagon mRNA expression levels. Treatment with liraglutide (50 and 100 *μ*g/kg) significantly reduced nicotine‐induced CPP scores, suggesting an attenuation of addictive behavior. Liraglutide downregulated the expression of prepro‐orexin and prepro‐glucagon, with the 100 *μ*g/kg dose demonstrating greater efficacy in normalizing prepro‐glucagon levels. These findings indicate that liraglutide mitigates nicotine reward by modulating neuropeptide pathways, including orexin and glucagon signaling. The dual impact of liraglutide on behavioral and molecular markers highlights its potential as a therapeutic agent for treating nicotine induce reward‐related behavior. Further research is warranted to explore its long‐term efficacy and underlying mechanisms in addiction modulation.

## 1. Introduction

Tobacco is known to affect human health significantly. It is reported that the worldwide consumption of tobacco products has increased dramatically in the last decade [1]. Cigarettes are one of the tobacco products that contribute to degenerative diseases such as lung cancer, pulmonary disease, and cardiovascular disease [2, 3]. Cigarettes contain over 7000 compounds, with more than 50 of those compounds having carcinogenic activity. According to the World Health Organization (WHO), cigarettes contribute to nearly 8 million mortalities, either for active or passive smokers [4].

Nicotine is the primary compound in cigarettes that causes addiction problems in smokers. This compound is suitable for penetrating the blood‐brain barrier and stimulates the central nervous system (CNS) [5, 6]. In CNS, the nicotine effect on the addiction is facilitated via stimulation of its receptor, the Nicotinic Acetylcholine receptors (nAChRs). There are several types of subunits of these receptors, including *α*4β2, *α*3β4, and *α*7. The activation of nAChRs due to its binding with nicotine caused an abundant release of dopamine, a neurotransmitter responsible for pleasure and mood feelings, from dopaminergic neurons. This dopamine release causes a reward effect, eventually leading to addictive behavior [7].

The addictive effect mainly occurs through the mesolimbic pathway, where the activation of nAChR receptors causes an increase in dopamine projection from the ventral tegmental area (VTA) to the Nucleus Accumbens (NAc). Continuous re‐exposure to nicotine leads to neuroadaptation and tolerance conditions. The increase of dopamine in the NAc results in the emergence of the reward effect [8]. This causes nAChRs to become sensitized, resulting in negative reinforcement. Once the dopamine transmission decreases due to the stopping of the nicotine administration, someone may experience discomfort feelings and prompts to seek out the addictive substance [9]. The effect of nicotine enhances cognitive function. Various researchers have shown that it is responsible for reinforcing behavioral change and nicotine dependence, where some nondopaminergic systems are also [10].

Nicotine influences various regions and impacts the expression of neurotransmitters regulated in these areas, such as the hypothalamus [11]. Besides dopamine, activation of nAChRs by nicotine increases the release of the neuropeptide orexin in the hypothalamus. This peptide is important in regulating behavioral cycles, energy balance, stress, arousal, and appetite [12, 13]. The activation of nAChRs increases the orexin projection to various areas, including the VTA [14]. Several studies have shown that nicotine administration activates orexin neurons, and blocking orexin transmission reduces nicotine‐seeking behavior. This indicates that orexin is involved in the process of nicotine addiction [15]. In addition, the VTA and NAc also project peptides such as GLP‐1 (Glucagon‐Like Peptide‐1) from the nucleus tractus solitarius (NTS) [16].

Nicotine increases cFos expression in the NTS, which helps regulate other gene expressions. This is related to the activation of nAChRs by nicotine through the activation of MAP‐kinases and CaM‐kinases [17]. In the context of cocaine addiction, cocaine administration increases cFos expression, influencing the activation of neurons in the NTS. This activity leads to an increase in prepro‐glucagon, the peptide precursor of GLP‐1. The elevation of GLP‐1 signaling following cocaine exposure acts as a homeostatic response to reduce further cocaine consumption. However, when cocaine use is discontinued, prepro‐glucagon expression in the NTS decreases, which triggers drug‐seeking behavior. This indicates that GLP‐1 derived from prepro‐glucagon functions primarily as a “brake” or defensive mechanism to mitigate further cocaine consumption [18].

Liraglutide is a GLP‐1 analog agonist commonly used to treat diabetes mellitus. It controls blood glucose levels by increasing insulin sensitivity, slowing gastric emptying, and reducing appetite [19]. One study showed that GLP‐1 agonists are capable of reducing nicotine self‐administration, suggesting that GLP‐1 agonists could be a potential treatment for individuals with addiction [20]. GLP‐1 receptors are G‐protein coupled receptors that work through the Gs pathway, activating cAMP. This activation subsequently affects the gamma‐aminobutyric acid (GABA) pathway, resulting in the inhibition of neurotransmitter activity in areas such as the hypothalamus and the VTA [21]. Considering the potential use of GLP‐1 agonists, the current study investigated the involvement of orexin and glucagon in the liraglutide (a GLP‐1 agonist) action to prevent nicotine‐induced addictive behavior.

## 2. Materials and Methods

### 2.1. Experimental Animals

**Figure 1:**
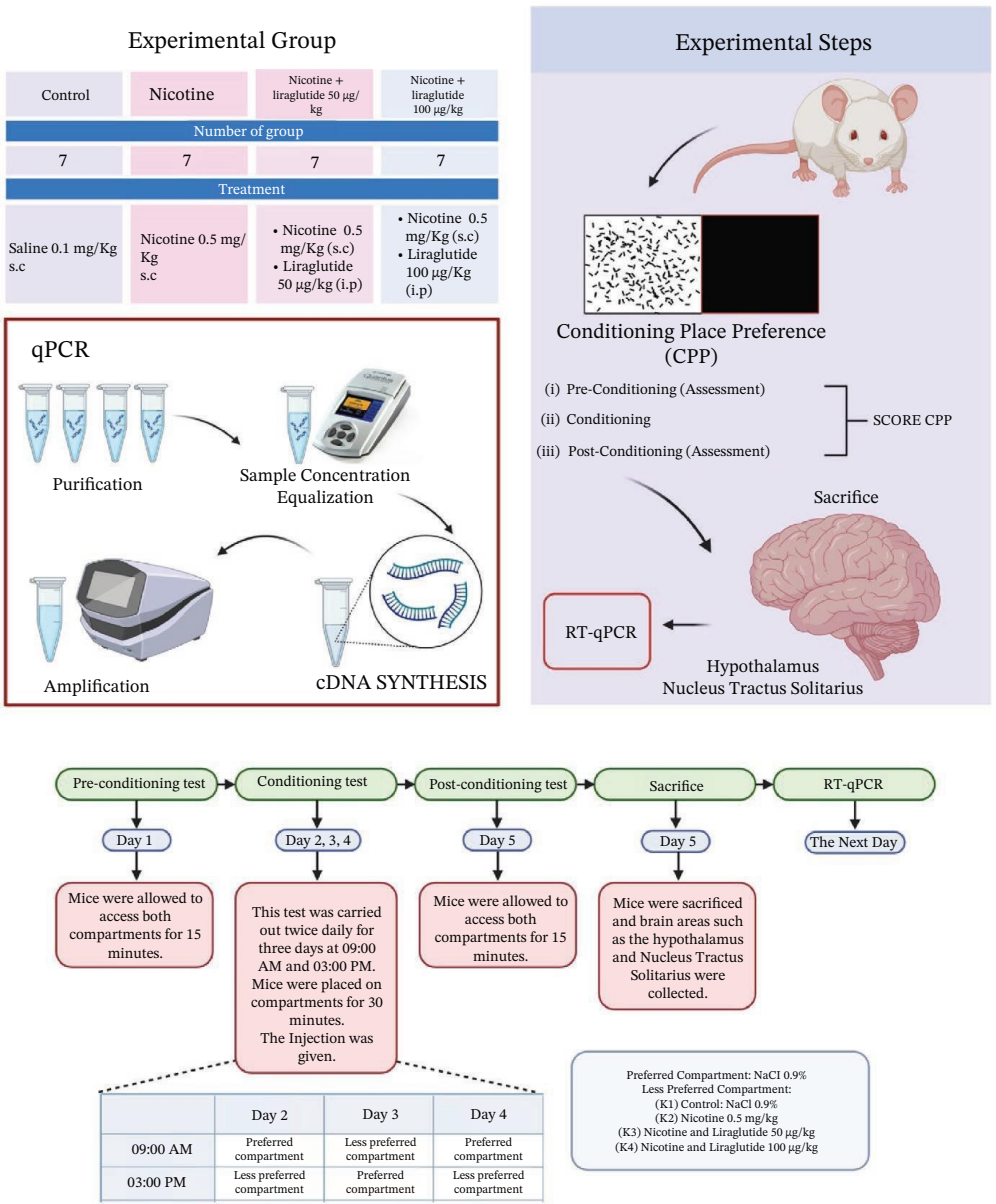
Experimental protocols. There were four groups (*n* = 7 per group), consisting of a control group, a nicotine group, and two treatment groups receiving liraglutide at doses of 50 and 100 *μ*g/kg, respectively. The conditioning place preference (CPP) paradigm was used as a model to evaluate the reward effects. The procedure consisted of three phases: preconditioning, assessment of the animals′ initial preference; conditioning, establishment of a conditioned association or perception; and postconditioning, assessment of preference after conditioning. Furthermore, RT‐qPCR analysis was performed to quantify gene expression levels in the hypothalamus and nucleus tractus solitarius (NTS).

All experiments were conducted at the Animal Laboratory Research Center, Faculty of Pharmacy, Universitas Airlangga, Surabaya, Indonesia. Ddy male mice (*Mus musculus*), 2–3 months old with a body weight of 20–30 g were used in this study. The animals will be excluded if animals die before treatment is completed, or if their body weight decreases > 20%. The mice were obtained from the Veterinary Center Surabaya, Indonesia. Mice were placed in cages (50 × 25 × 20 cm) with a temperature of 24^°^C ± 1^°^C and a 12/12 h light and dark cycle. Mice were divided into four groups and acclimatized for 7 days before the experiment. All protocols (Figure 1) were approved by the research ethics committee of the Faculty of Veterinary Medicine, Universitas Airlangga, Surabaya, Indonesia. This research has been declared ethically sound as evidenced by the ethical suitability Certificate Number: 2.KEH.057.04.2024.

### 2.2. Drugs and Experimental Design

Nicotine barbiturate dihydrate (*Tokyo Chemical Industry*) with 162.24 g/mol molecular weight, liraglutide 6 mg/mL (Victoza, Novo Nordisk), and NaCl 0.9% (PT. Otsuka Indonesia) were used in this research. Nicotine and liraglutide were dissolved in normal saline (NaCl 0.9%) in a recenter apparatus. The mice were randomly divided into four groups with simple randomization, each with seven mice, based on Federer′s formula: (1) Normal group, mice were injected with normal saline 1 mL/100 g subcutaneously (s.c.) 2 times daily for 3 days; (2) nicotine group, mice were injected with nicotine 0.5 mg/kg s.c one time daily and normal saline 1 mL/100 g subcutaneously (s.c.) one time daily for 3 days in morning and afternoon alternately; (3) nicotine + liraglutide 50 *μ*g/kg, mice were injected with nicotine 0.5 mg/kg s.c one time daily after 30 min injected with liraglutide 50 *μ*g/kg intraperitoneal (i.p.) and normal saline 1 mL/100 g subcutaneously (s.c.) one time daily after 30 min injected with normal saline 1 mL/100 g i.p. for 3 days in morning and afternoon alternately; (4) nicotine + liraglutide 100 *μ*g/kg, mice were injected with nicotine 0.5 mg/kg s.c one time daily after 30 min injected with liraglutide 100 *μ*g/kg i.p. and normal saline 1 mL/100 g subcutaneously (s.c.) one time daily after 30 min injected with normal saline 1 mL/100 g i.p. for 3 days in morning and afternoon alternately. No blinding was performed during the allocation, conduct, outcome assessment, or data analysis.

### 2.3. Nicotine Reward and Aversive Effects Examinations

Conditioning place preference (CPP) was used to measure the rewarding (positive) or aversive (negative) effects of substances with boxes in two different compartments (white with a rough texture and black with a smooth texture) separated by a partition. A positive affective response is defined when a subject spends more time in an environment where it previously received a rewarding substance, such as nicotine. Conversely, spending less time in an environment where it received an aversive substance indicates a negative affective response. This method uses three designs: preconditioning, conditioning, and postconditioning. (1) Preconditioning: Mice were placed in boxes without partitions to explore two compartments within 15 min (2 days). This was done to find out which compartment the mice preferred. The time duration was calculated and recorded with a stopwatch according to the time the mice spent in each compartment. (2) Conditioning: Mice were placed in boxes with partitions within 45 min (3 days) and carried out in the morning (09:00 a.m) and afternoon (01:30 p.m). Mice were injected with drugs, according to the group. Saline was injected for preferred compartments, and nicotine was injected for unpreferred compartments. For groups that give treatment, liraglutide was injected 30 min before nicotine. Normal groups were injected with saline in two compartments. This is to challenge mice in an area they do not prefer by administering nicotine to determine the reward that occurs. In the morning, mice were injected with saline and placed in a preferred compartment within 45 min; in the afternoon, mice were injected with nicotine (with or without liraglutide) and placed in an unpreferred compartment within 45 min. Next days, In the morning, mice were injected nicotine (with or without liraglutide) and placed in an unpreferred compartment within 45 min; in the afternoon, mice were injected with saline and placed in a preferred compartment within 45 min. The next day is the same as the first day of conditioning. (3) Postconditioning: Mice were placed in boxes without partitions to explore two compartments within 15 min (1 day). This was done to find out which compartment the mice preferred after conditioning. It is the same as preconditioning. These experiments recorded the time mice spent and calculated within a score (postconditioning–preconditioning) in seconds.

### 2.4. Assessment of Prepro‐Orexin and Prepro‐Glucagon

At the end of the study, mice were sacrificed to assess orexin and glucagon signaling in the brain. Animals were euthanized by cervical dislocation performed by trained personnel in accordance with institutional animal care guidelines. Mice′s hypothalamus and NTS were sliced using a brain blocker according to the mice′s brain atlas. Brain samples were immediately frozen in liquid nitrogen and stored at −80°C. Reverse transcription quantitative polymerase chain reaction (RT‐qPCR) was used to assess the expression of prepro‐orexin mRNA in the hypothalamus and prepro‐glucagon in NTS. The total RNA of both samples was extracted using the Total RNA Purification Kit (Jena Bioscience). The synthesis of cDNA with reverse transcription was conducted using the GoScriptTM Reverse Transcription System (Promega, United States). GoTaq qPCR master mix (Promega, United States) was used to amplify the primer target (as shown in Table 1). The temperature setting for amplification was based on the GoTaq qPCR master mix guidelines. Melting temperature analysis after amplification was used to confirm the specificity of the PCR product. The mRNA expression was calculated using the 2‐*ΔΔ*CT formula.

**Table 1:** The primer sequence of the prepro‐orexin, prepro‐glucagon, and *β*‐actin.

Genes	Primer sequence	PCR product
Prepro‐orexin F	5 ^′^‐TGTTCCTGCCGTCTCTACGAA‐3 ^′^	137 bp
Prepro‐orexin R	5 ^′^‐TGGTTACCGTTGGCCTGAA‐3 ^′^	
Prepro‐glucagon F	5 ^′^‐TGAAGACAAACGCCACTCAC‐3 ^′^	132 bp
Prepro‐glucagon R	5 ^′^‐TGACGTTTGGCAATGTTGTT‐3 ^′^	
*β*‐actin F	5 ^′^‐TTCTTGGGTATGGAATCCTGT3 ^′^	101 bp
*β*‐actin R	5 ^′^‐AGCACTGTGTTGGCATAGAG‐3 ^′^	

### 2.5. Data Analysis

GraphPad Prism ver 9.1 (GraphPad, Inc., San Diego, California, United States) software was used for statistical analysis. Data were analyzed using the one‐way analysis of variance (ANOVA) method, followed by Tukey′s post hoc test. *p* < 0.05 was considered significant. No animals or data points were excluded from the analysis.

## 3. Results

### 3.1. Liraglutide Ameliorates Nicotine Reward‐Related Behavior

**Figure 2:**
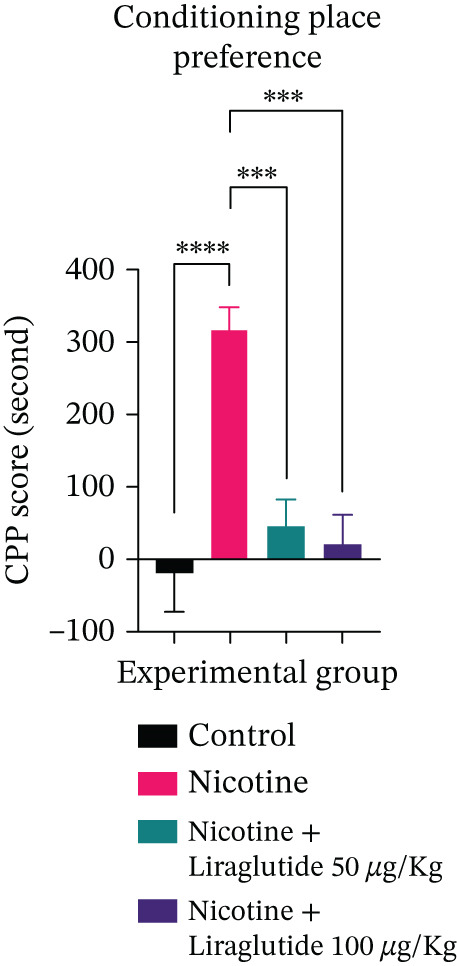
CPP scores for each treatment group. Data are presented as mean ± SEM (seconds) and analyzed using one‐way ANOVA with post hoc Tukey′s test.  ^∗∗∗^ *p* < 0.001 Nicotine versus nicotine liraglutide 50 mg/kg;  ^∗∗∗^ *p* < 0.001 nicotine versus nicotine liraglutide 100 mg/kg;  ^∗∗∗∗^ *p* < 0.0001 control versus nicotine.

The CPP model is a widely accepted method for studying reward‐related behaviors. It refers to the ability to associate a stimulus with a specific period or context. This model integrates learning, memory, and motivated behavior. One study demonstrated that the use of this model effectively revealed the reward condition associated with nicotine addiction and its significant reduction following treatment [22, 23]. This study employed the CPP model to evaluate reward‐related behaviors induced by nicotine. In previous research, nicotine 0.5 mg/kg showed efficacy in causing reward in the CPP test [24]. Based on this study, nicotine 0.5 mg/kg significantly caused reward in the normal group, and liraglutide affected the reward, which reduced reward‐related behavior. This indicates that liraglutide is capable of reducing the reward effects induced by nicotine administration, as evidenced by a significant decrease in CPP scores compared with the nicotine‐treated group. The administration of liraglutide 50 and 100 *μ*g/kg significantly reduced reward to nicotine from the score of CPP (Figure 2). This is indicated by a *p* value of < 0.001, showing a statistically significant difference compared with the nicotine‐treated group.

### 3.2. Liraglutide Decreases the mRNA Expressions of Prepro‐Orexin in Hypothalamus and Prepro‐Glucagon in NTS

**Figure 3:**
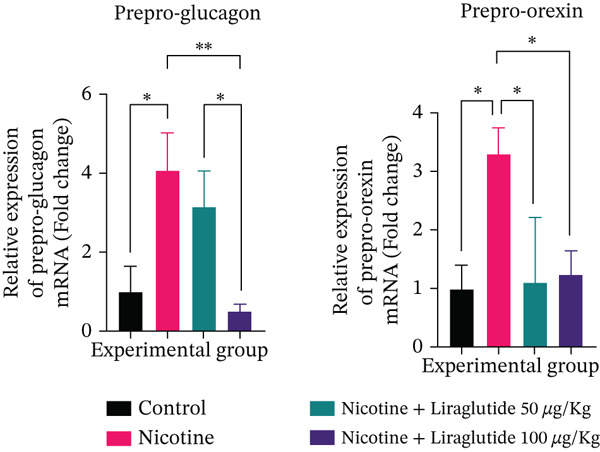
Relative mRNA expression of prepro‐orexin (fold change) in the hypothalamus and prepro‐glucagon (fold change) in the NTS, measured using real‐time PCR and analyzed with one‐way ANOVA with post hoc Tukey′s test.  ^∗^ *p* < 0.05;  ^∗∗^ *p* < 0.01.

In this study, the mRNA expression of the prepro‐orexin and prepro‐glucagon genes was measured using the RT‐qPCR method. The analysis focused on the NTS and hypothalamus, brain regions associated with the effects of nicotine on the expression of these genes, which are implicated in reward. RT‐qPCR analysis was performed after the animals underwent the CPP treatment, followed by dissection to collect the NTS and hypothalamus brain regions for analysis in each experimental group. Nicotine injection of 0.5 mg/kg significantly increased the levels of prepro‐orexin mRNA in the hypothalamus and prepro‐glucagon in NTS. Meanwhile, the administration of liraglutide 50 and 100 *μ*g/kg significantly reduced the levels of prepro‐orexin mRNA in the hypothalamus and prepro‐glucagon in NTS (Figure 3).

## 4. Discussion

This study demonstrated that administering nicotine at a dose of 0.5 mg/kg induces a reward effect in experimental animals. This finding aligns with a previous study [25], which showed that 0.5 mg/kg of nicotine elicited reward effects in experimental animals, as evidenced by the CPP method that revealed increased aversive scores. In this research, nicotine administration at 0.5 mg/kg resulted in a significant CP*P* value (*p* < 0.0001) compared with the control group, indicating that the test animals experienced reward effects at this dose.

Furthermore, this study also evaluated the effects of treatment with liraglutide at two different doses, 50 and 100 *μ*g/kg. Liraglutide, as a GLP‐1 receptor agonist, is known to influence the CNS and modulate the reward effects of addictive substances. The role of liraglutide in this context is characterized by its negative impact on the reward effects of addictive agents. Neurotransmitter signaling influenced by liraglutide along specific pathways reduces the reward effects. A study by Tuesta et al. [20] supports these findings, showing that GLP‐1 receptor activation reduces nicotine reward, decreasing nicotine use, whereas knockdown of GLP‐1 receptors increases nicotine use. In this study, liraglutide treatment at doses of 50 and 100 *μ*g/kg reduced reward scores associated with nicotine reward. As shown in Figure 2, CPP scores decreased significantly in the treatment groups, with both doses showing significance at *p* < 0.001 compared with the nicotine‐only group. This demonstrates that liraglutide at these doses effectively diminishes the reward effects of nicotine, indicating its inhibitory effect on the CNS pathways regulating reward in the brain.

**Figure 4:**
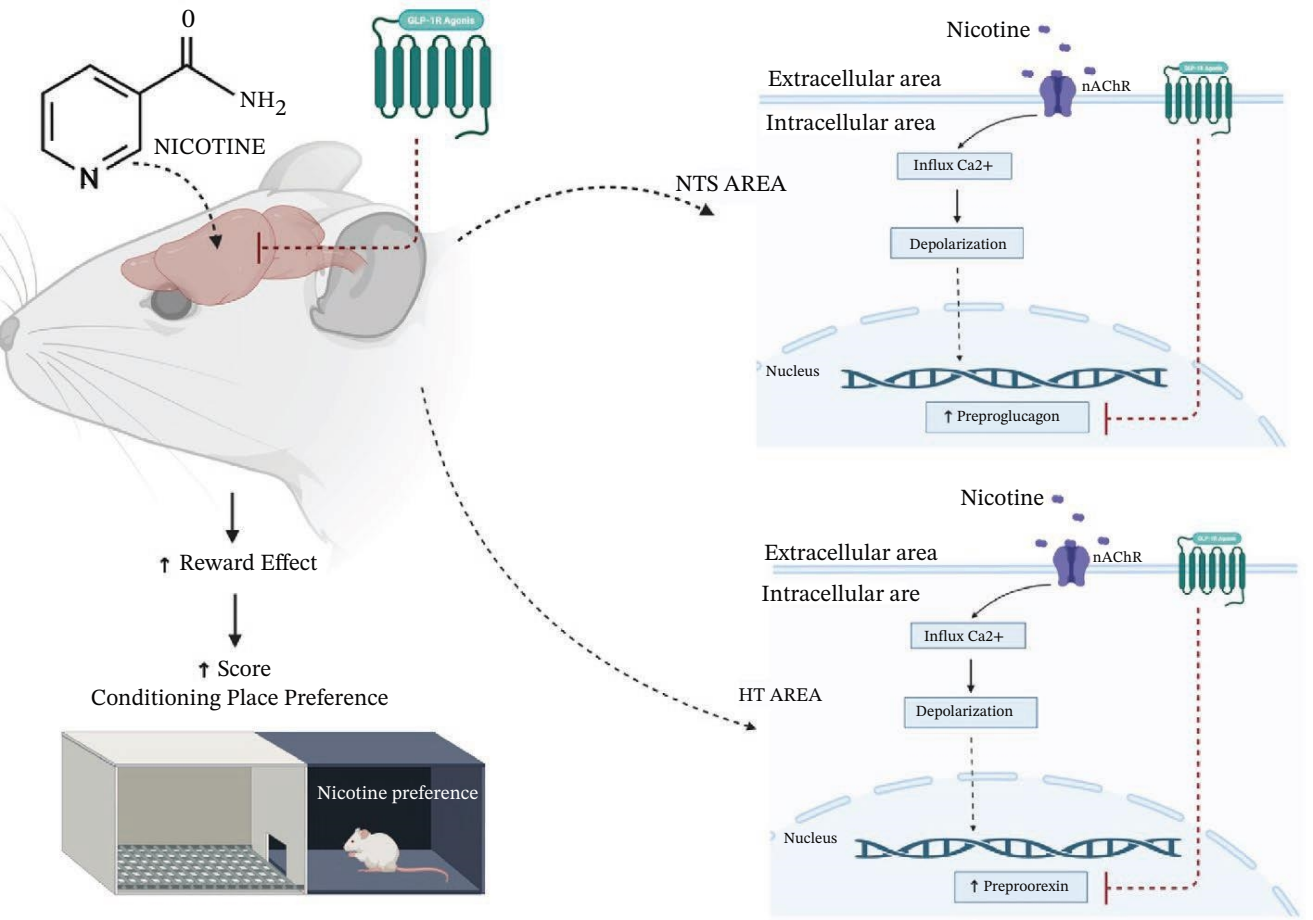
The activation of nAChR by leading to an increase in reward effects as observed through CPP scores. Nicotine activates nicotinic acetylcholine receptors (nAChRs) on neuronal cell membranes. This activation causes an influx of Ca^2+^ and depolarization of the neuron, resulting in the activation of gene transcription in the nucleus. It is an increase in prepro‐orexin in the hypothalamus (HT) and elevated prepro‐glucagon levels in the NTS, which are also associated with reward effects. GLP‐1 agonists reduce the reward effects produced.

In general, as shown in Figure 4, reward effects in the CNS are mediated by dopamine neurotransmitters from the VTA, resulting in reward sensations. This pathway is innervated by the NAc, which enhances dopamine expression in the VTA through nAChRs [8]. Other research has indicated that this effect is also mediated by neurotransmitters such as prepro‐glucagon. The present study found that nicotine significantly increases the prepro‐glucagon mRNA expression in the NTS. In line with the present result, a study by Tuesta et al. [20] revealed that nicotine intake activates the GLP‐1 neuron signaling in the NTS. The activation of this signaling has a reverse regulation role of nicotine since it suppresses the nicotine reward effect. Considering the critical role of GLP‐1 on the nicotine reward effect, this suggests that the increase of prepro‐glucagon expression in this present study is a natural negative feedback mechanism of the mice to suppress the reward effect of nicotine. The mechanism involved acts as a sensor that is activated and triggers an avoidance circuit in response to nicotine consumption. This serves as the brain′s warning system against high doses of nicotine through the activation of GLP‐1 receptors. The present study′s liraglutide treatment ameliorated the nicotine‐induced prepro‐glucagon mRNA overexpression in the NTS. This result suggests that the negative effects of nicotine reward in mice were normalized by liraglutide treatment. This condition represents a neuroadaptive response aimed at maintaining homeostasis against excessive nicotine consumption [20, 26]. The decrease of prepro‐glucagon mRNA expression in the NTS possibly occurs due to the direct activation of GLP‐1 receptors by liraglutide in several neurons that received projection from NTS’s GLP‐1 neurons.

In the present study, the reduction of prepro‐glucagon expression only appears in the liraglutide dose 100 *μ*g, but not in the 50 *μ*g treatment group. This result indicates that at a dose of 100 *μ*g, liraglutide is capable of externally enhancing GLP‐1 signaling. Endogenous GLP‐1 signaling within the mesolimbic structure contributes to the regulation of reward‐related motivation. Prepro‐glucagon, as the precursor of GLP‐1, functions in a feedback regulatory manner—where increased GLP‐1 serves as an avoidance circuit to alert the brain to high nicotine doses. The role of GLP‐1R agonists does not completely replace the endogenous GLP‐1 system; rather, both interact with existing GLP‐1 neuronal circuits to modulate activity and expression patterns. When GLP‐1R agonists are administered at high doses and prepro‐glucagon levels decrease to the control level, this suggests the presence of a negative feedback mechanism. The brain responds to strong GLP‐1R activation by reducing endogenous GLP‐1 production. This condition reflects a homeostatic mechanism within the body that regulates excessive modulation of neuronal activity and gene expression [20]. The strong and rapid effects of nicotine cause imbalances in the neurotransmitter system, allowing nicotine to continue causing reward. The brain responds directly by initiating a negative feedback mechanism to restore balance under conditions of excessive exposure. One of the regulatory systems involved when nicotine exposure becomes excessive is the GLP‐1 pathway. This aligns with the administration of 50 and 100 *μ*g doses of liraglutide. At the 50 *μ*g dose, the system still requires higher GLP‐1 expression to balance neurotransmitter regulation and reduce reward‐related behavior. Meanwhile, at the 100 *μ*g dose, the body has sufficiently utilized the dose to modulate neurotransmitter balance, resulting in reduced dependency on additional production of GLP‐1. The observed decrease serves as a signal from the brain indicating that GLP‐1R activation has reached a sufficient level to modulate the reward effects induced by nicotine. The body strives to maintain equilibrium in regulating neurotransmitters, particularly in response to reward effects [27].

There is evidence of a unique neural pathway system in the projection of increased reward effects caused by nicotine, linked with other neurotransmitters involved in liraglutide treatment. The neurotransmitter projections to reward‐related areas depend on the receptors present in those regions. Nicotine has been shown to enhance orexin expression in the hypothalamus due to the presence of nAChRs in the hypothalamus, increasing orexin levels [28]. Upregulation of the orexin signaling in the hypothalamus is important since the hypothalamus contains a cell body of orexin neurons that is projected to several brain areas [29]. Consistent with previous findings, blockade of orexin‐1 receptor signaling attenuates nicotine‐induced reward and cue‐induced reinstatement of nicotine‐seeking behavior [27]. This evidence suggests that the effect of nicotine administration on several brain areas may be facilitated by activating orexin signaling. In the present study, administering liraglutide in all doses ameliorated the overexpression of prepro‐orexin in the hypothalamus.

Orexin acts as one of the mediators involved in drug‐seeking behavior. Activation of orexin in the NAc shows a positive correlation with the increased seeking of addictive substances. Blocking orexin signaling in the NAc has been shown to reduce reward effects, as observed in negative control experiments. This indicates that orexin also plays an important role in modulating dopamine release and the reward circuit, and it has the potential to serve as a target for suppressing addictive behaviors [30]. This result aligns with the study by Acuna‐Goycolea and van den Pol, 2004 that shows the direct effect of GLP‐1 activation on the hypothalamic orexin neuron polarization. Recovery from nicotine seeking indicates a specific role of individual neurons in conditions affected by nicotine administration. Nicotine administration has been shown to increase the expression of preorexin, which originates from the prepro‐orexin transcript in the hypothalamus. A study by Hollander et al. (2008) demonstrated a relationship between orexin administration and the reward effect, where inhibition of orexin receptors reduces nicotine self‐administration. This is further supported by other research showing that increased orexin levels positively correlate with enhanced dopamine release in the NAc, which is induced by addictive substances [31]. These findings suggest that orexin plays a significant role in the reinforcement of addictive behaviors and reward processing. Results from the present study significantly demonstrate that liraglutide administration is capable of restoring the orexin expression disturbance and maintaining behavioral preference in mice towards nicotine stimuli.

The activation of orexin neurons may be attributed to increased motivation and attention processing involved in nicotine relapse. Treatment with SB‐334867, an OX1R antagonist, before naloxone‐induced withdrawal was found to reduce the severity of somatic withdrawal manifestations. Moreover, the intensity of these withdrawal symptoms was positively associated with cFos expression in the NAc shell. Inhibition of OX1R appeared to indirectly suppress neuronal activation within the NAc. Notably, no significant alterations in cFos expression were observed in the VTA of the same animals, indicating that orexin‐mediated modulation of NAc activity may occur through mechanisms independent of the VTA [31]. The present study demonstrates that GLP‐1 receptor agonists influence nicotine‐associated reward by regulating the prepro‐orexin and prepro‐glucagon signaling pathways. In humans, these two pathways are recognized as key contributors to the control of motivation and reward‐related behaviors. Clinical evidence further supports this relationship; for example, a study by Al′Absi et al. [32] reported that orexin levels are associated with cigarette craving in smokers. In addition, clinical investigations have shown that GLP‐1 receptor agonists reduce alcohol craving in individuals with addictive disorders [19, 32]. These findings suggest that our results obtained in mice are consistent with clinical observations, highlighting the inhibitory role of GLP‐1 receptor activation in reward processing. Further clinical studies are warranted to strengthen the evidence supporting the involvement of these systems in addiction and metabolic regulation in humans. Although rodent models cannot fully replicate the psychosocial complexity of nicotine dependence in humans, the conserved molecular and circuit‐level mechanisms reinforce the translational relevance of our findings.

The results of this study indicate that nicotine‐induced reward‐related behavior leads to dysregulation across various neurotransmitter pathways, which affects other conditions beyond just reward. This is evident in how reward conditions influence the projections of prepro‐orexin and prepro‐glucagon. The alterations in these pathways highlight the complex interplay between reward or addiction and the regulation of different neuropeptides, suggesting that nicotine′s impact extends to various neurobiological systems, influencing not only reward but also other physiological processes. Although these findings are promising, this study has certain limitations. The researchers did not analyze the direct effect of liraglutide administration in inducing the CPP paradigm to find out whether there may be any intrinsic rewarding or aversive effects of liraglutide itself in the CPP paradigm. Nicotine administration has been shown to increase prepro‐glucagon expression. However, treatment with GLP‐1 agonists (such as liraglutide), exendin‐4, or sitagliptin reduces nicotine‐induced reward effects. These findings suggest a potential association between decreased prepro‐glucagon expression and the attenuation of nicotine reward. Further studies are required to elucidate the underlying mechanisms of this relationship. Furthermore, these data could serve as a foundation for future investigations into this pathway as a potentially promising target.

## 5. Conclusions

This study shows the ameliorated effect of liraglutide on the nicotine‐induced reward‐related behavior caused by nicotine. The effect of liraglutide was possibly caused by the regulation of prepro‐orexin in the hypothalamus and prepro‐glucagon in the NTS.

## Author Contributions

M.R.: conceptualization (lead), data curation (lead), formal analysis (equal), methodology (lead), resources (lead), supervision (equal), validation (equal), writing—review and editing (equal), funding acquisition (lead), and software (lead). C.A.: conceptualization (equal), methodology (equal), project administration (lead), supervision (equal), formal analysis (equal), validation (equal), and writing—review and editing (equal). R.R.: formal analysis (equal), methodology (equal), supervision (equal), validation (equal), and writing—review and editing (supporting). D.A.: data curation (equal), investigation (equal), project administration (equal), resources (equal), software (equal), visualization (lead), and writing—original draft (lead). A.P.S.A.F.: data curation (equal), investigation (equal), project administration (equal), resources (equal), and software (equal). I.N.B.S.: investigation (supporting), supervision (equal), writing—review and editing (equal), and software (equal). A.D.N.: investigation (supporting), supervision (equal), writing—review and editing (equal), and software (equal).

## Funding

This research was funded by the Universitas Airlangga under the scheme of Airlangga Research Fund 2024 (International Research Collaboration Top #300 2024) (No. 413/UN3.LPPM/PT.01.03/2024).

## Conflicts of Interest

The authors declare no conflicts of interest.

## Data Availability

The data that support the findings of this study are available from the corresponding author upon reasonable request.

## References

[1] O′Connor R. , Watson C. H. , Swan G. E. , Nettles D. S. , Geisler R. C. , Hendershot T. P. , and PhenX TRR Agent Working Group , PhenX: Agent Measures for Tobacco Regulatory Research, Tobbaco Control. (2020) 29, no. Supplement 1, s20–s26, 10.1136/tobaccocontrol-2019-054976, 31992660. PMC8127628 31992660

[2] Jha P. , The Hazards of Smoking and the Benefits of Cessation: A Critical Summation of the Epidemiological Evidence in High-Income Countries, eLife. (2020) 9, e49979, 10.7554/eLife.49979. 32207405 PMC7093109

[3] Omari M. O. , Kibet J. K. , Cherutoi J. K. , Bosire J. O. , and Rono N. K. , Heavy Metal Content in Mainstream Cigarette Smoke of Common Cigarettes sold in Kenya, and Their Toxicological Consequences, International Research Journal of Environment Sciences. (2015) 4, no. 6, 75–79.

[4] Omare M. O. , Kibet J. K. , Cherutoi J. K. , and Kengara F. O. , A Review of Tobacco Abuse and Its Epidemiological Consequences, Journal of Public Health. (2022) 30, no. 6, 1485–1500, 10.1007/s10389-020-01443-4, 33425659. 33425659 PMC7786188

[5] National Cancer Institute , Harms of Cigarette Smoking and Health Benefits of Quitting, 2017, U.S. Department of Health Human Services, National Institutes of Health.

[6] Singh N. , Wanjari A. , and Sinha A. H. , Effects of Nicotine on the Central Nervous System and Sleep Quality in Relation to Other Stimulants: A Narrative Review, A Narrative Review, Cureus. (2023) 15, no. 11, 10.7759/cureus.49162. PMC10733894 38130519

[7] Herman A. I. , DeVito E. E. , Jensen K. P. , and Sofuoglu M. , Pharmacogenetics of Nicotine Addiction: Role of Dopamine, Pharmacogenomics. (2014) 15, no. 2, 221–234, 10.2217/pgs.13.246, 24444411. 24444411 PMC4154357

[8] Addicott M. A. , Sweitzer M. M. , and McClernon F. J. , The Effects of Nicotine and Tobacco Use on Brain Reward Function: Interaction With Nicotine Dependence Severity, Nicotine & Tobacco Research. (2019) 21, no. 6, 764–771, 10.1093/ntr/nty059, 29584917. 29584917 PMC6784410

[9] Benowitz N. L. , Nicotine Addiction, New England Journal of Medicine. (2010) 362, no. 24, 2295–2303, 10.1056/NEJMra0809890, 20554984. 20554984 PMC2928221

[10] Tiwari R. K. , Sharm V. , Pandey R. K. , and Shukla S. S. , Nicotine Addiction: Neurobiology and Mechanism, Journal of Pharmacopuncture. (2020) 23, no. 1, 1–7, 10.3831/KPI.2020.23.001, 32322429. 32322429 PMC7163392

[11] Rahmadi M. , Nurhan A. D. , Rahmawati R. I. A. , Damayanti T. F. , Purwanto D. A. , and Khotib J. , Epigallocatechin Gallate Ameliorates Nicotine Withdrawal Conditions-Induced Somatic and Affective Behavior Changes in Mice and Its Molecular Mechanism, Behavioural Neurology. (2023) 2023, 5581893, 10.1155/2023/5581893, 37346971. 37346971 PMC10281828

[12] Wang C. , Wang Q. , Ji B. , Pan Y. , Xu C. , Cheng B. , Bai B. , and Chen J. , The Orexin/Receptor System: Molecular Mechanism and Therapeutic Potential for Neurological Diseases, Frontiers in Molecular Neuroscience. (2018) 11, 10.3389/fnmol.2018.00220, 30002617. PMC6031739 30002617

[13] Inutsuka A. and Yamanaka A. , The Physiological Role of Orexin/Hypocretin Neurons in the Regulation of Sleep/Wakefulness and Neuroendocrine Functions, Frontiers in Endocrinology. (2013) 4, 10.3389/fendo.2013.00018, 23508038. PMC3589707 23508038

[14] Yon-Seng Khoo S. , McNally G. P. , and Clemens K. J. , The Orexin System and Nicotine Addiction: Preclinical Insights, 2019, Academic Press: Neuroscience of Nicotine.

[15] Hollander J. A. , Lu Q. , Cameron M. D. , Kamenecka T. M. , and Kenny P. J. , Insular hypocretin Transmission Regulates Nicotine Reward, Proceedings of National Academy of Sciences of the USA. (2008) 105, no. 49, 19480–19485, 10.1073/pnas.0808023105, 19033203. PMC2614786 19033203

[16] Hernandez N. S. , Weir V. R. , Ragnini K. , Merkel R. , Zhang Y. , Mace K. , Rich M. T. , Christopher Pierce R. , and Schmidt H. D. , GLP-1 Receptor Signaling in the Laterodorsal Tegmental Nucleus Attenuates Cocaine Seeking by Activating GABAergic Circuits That Project to the VTA, Molecular Psychiatry. (2021) 26, no. 8, 4394–4408, 10.1038/s41380-020-00957-3, 33257815. 33257815 PMC8164646

[17] Sherafat Y. , Bautista M. , and Fowler C. D. , Multidimensional Intersection of Nicotine, Gene Expression, and Behavior, Frontiers in Behavioral Neuroscience. (2021) 15, 10.3389/fnbeh.2021.649129, 33828466. PMC8019722 33828466

[18] Schmidt H. D. , Mietlicki-Baase E. G. , Ige K. Y. , Maurer J. J. , Reiner D. J. , Zimmer D. J. , van Nest D. S. , Guercio L. A. , Wimmer M. E. , Olivos D. R. , de Jonghe B. C. , and Hayes M. R. , Glucagon-Like Peptide-1 Receptor Activation in the Ventral Tegmental Area Decreases the Reinforcing Efficacy of Cocaine, Neuropsychopharmacology : Official Publication of the American College of Neuropsychopharmacology. (2016) 41, no. 7, 1917–1928, 10.1038/npp.2015.362, 26675243. 26675243 PMC4869061

[19] Klausen M. K. , Thomsen M. , Wortwein G. , and Fink-Jensen A. , The Role of Glucagon-Like Peptide 1 (GLP-1) in Addictive Disorders, British Journal of Pharmacology. (2022) 179, no. 4, 625–641, 10.1111/bph.15677, 34532853. 34532853 PMC8820218

[20] Tuesta L. M. , Chen Z. , Duncan A. , Fowler C. D. , Ishikawa M. , Lee B. R. , Liu X. A. , Lu Q. , Cameron M. , Hayes M. R. , Kamenecka T. M. , Pletcher M. , and Kenny P. J. , GLP-1 Acts on Habenular Avoidance Circuits to Control Nicotine Intake, Nature Neuroscience. (2017) 20, no. 5, 708–716, 10.1038/nn.4540, 28368384. 28368384 PMC5541856

[21] Farkas I. , Vastagh C. , Farkas E. , Bálint F. , Skrapits K. , Hrabovszky E. , Fekete C. , and Liposits Z. , Glucagon-Like Peptide-1 Excites Firing and Increases GABAergic Miniature Postsynaptic Currents (mPSCs) in Gonadotropin-Releasing Hormone (GnRH) Neurons of the Male Mice via Activation of Nitric Oxide (NO) and Suppression of Endocannabinoid Signaling Pathways, Frontiers in Cellular Neuroscience. (2016) 10, 10.3389/fncel.2016.00214. PMC5018486 27672360

[22] McKendrick G. and Graziane N. M. , Drug-Induced Conditioned Place Preference and Its Practical Use in Substance Use Disorder Research, Frontiers in Behavioral Neuroscience. (2020) 14, 10.3389/fnbeh.2020.582147, 33132862. PMC7550834 33132862

[23] Samirah S. , Indrayanti K. , Nurhan A. D. , Softyana V. , Pradipta N. , Ardianto C. , Yulistiani Y. , and Rahmadi M. , Andrographolide and Epigallocatechin Gallate (EGCG) Lower the Risk of Addiction Induced by Nicotine and Cigarette Smoke Extract (CSE) in Mice, Pharmaceutical Sciences. (2022) 28, no. 4, 10.34172/PS.2022.2.

[24] Kota D. , Martin B. R. , Robinson S. E. , and Damaj M. I. , Nicotine Dependence and Reward Differ Between Adolescent and Adult Male Mice, Journal Pharmacology and Experimental Therapeutics. (2007) 322, no. 1, 399–440, 10.1124/jpet.107.121616. 17446302

[25] Ahsan H. M. , de la Peña J. B. , Botanas C. J. , Kim H. J. , Yu G. Y. , and Cheong J. H. , Conditioned Place Preference and Self-Administration Induced by Nicotine in Adolescent and Adult Rats, Biomolecules & Therapeutics. (2014) 22, no. 5, 460–466, 10.4062/biomolther.2014.056, 25414778. 25414778 PMC4201227

[26] Sandoval D. A. and D′Alessio D. A. , Physiology of Proglucagon Peptides: Role of Glucagon and GLP-1 in Health and Disease, American Physiological Society. (2015) 95, no. 2, 513–548, 10.1152/physrev.00013.2014, 25834231. 25834231

[27] Plaza-Zabala A. , Flores Á. , Martín-García E. , Saravia R. , Maldonado R. , and Berrendero F. , A Role for Hypocretin/Orexin Receptor-1 in Cue-Induced Reinstatement of Nicotine-Seeking Behavior, Neuropsychopharmacology. (2013) 38, no. 9, 1724–1736, 10.1038/npp.2013.72, 23518606. 23518606 PMC3717542

[28] Kang X. , Tang H. , Liu Y. , Yuan Y. , and Wang M. , Research Progress on the Mechanism of Orexin in Pain Regulation in Different Brain regions, Life Sciences. (2021) 16, no. 1, 46–52, 10.1515/biol-2021-0001, 33817297. PMC7874592 33817297

[29] Acuna-Goycolea C. and van den Pol A. , Glucagon-Like Peptide 1 Excites Hypocretin/Orexin Neurons by Direct and Indirect Mechanisms: Implications for Viscera-Mediated Arousal, Journal of Neuroscience. (2004) 24, no. 37, 8141–8152, 10.1523/JNEUROSCI.1607-04.2004, 15371515. 15371515 PMC6729787

[30] Baimel C. , Lau B. K. , Qiao M. , and Borgland S. L. , Projection-Target-Defined Effects of Orexin and Dynorphin on VTA Dopamine Neurons, Cell Reports. (2017) 18, no. 6, 1346–1355, 10.1016/j.celrep.2017.01.030, 28178514. 28178514

[31] Sharf R. , Sarhan M. , and Dileone R. J. , Role of Orexin/Hypocretin in Dependence and Addiction, Brain Research. (2010) 1314, 130–138, 10.1016/j.brainres.2009.08.028, 19699189. 19699189 PMC2819591

[32] al′Absi M. , Lemieux A. , Hodges J. S. , and Allen S. , Circulating Orexin Changes During Withdrawal Are Associated With Nicotine Craving and Risk for Smoking Relapse, Addiction Biology. (2019) 24, no. 4, 743–753, 10.1111/adb.12643, 30117237. 30117237 PMC7263380

